# Intravascular versus surface cooling for targeted temperature management after out-of-hospital cardiac arrest: an analysis of the TTH48 trial

**DOI:** 10.1186/s13054-019-2335-7

**Published:** 2019-02-22

**Authors:** Chiara De Fazio, Markus B. Skrifvars, Eldar Søreide, Jacques Creteur, Anders M. Grejs, Jesper Kjærgaard, Timo Laitio, Jens Nee, Hans Kirkegaard, Fabio Silvio Taccone

**Affiliations:** 1 Department of Intensive Care, Cliniques Universitaires de Bruxelles Hopital Erasme, Université Libre de Bruxelles (ULB), Route de Lennik, 808, 1070 Brussels, Belgium; 2 Division of Intensive Care, Department of Anesthesiology, Intensive Care and Pain Medicine, University of Helsinki and Helsinki University Hospital, Helsinki, Finland; 3 Critical Care and Anaesthesiology Research Group, Stavanger University Hospital, Stavanger, Norway; 4 Department of Clinical Medicine, University of Bergen, Bergen, Norway; 5 Department of Intensive Care Medicine, Aarhus University Hospital, Aarhus, Denmark; 6 Department of Cardiology B, Copenhagen University Hospital Rigshospitalet, Copenhagen, Denmark; 7 Division of Perioperative Services, Intensive Care Medicine and Pain Management, Turku University Hospital, Turku, Finland; 8 Medizinische Klinik mit Schwerpunkt, Nephrologie und Internistische Intensivmedizin, Charité-Universitätsmedizin Berlin, Berlin, Germany; 9 Research Center for Emergency Medicine, Department of Emergency Medicine and Department of Clinical Medicine, Aarhus University Hospital and Aarhus University, Aarhus, Denmark; 10 Department of Emergency Care and Services, University of Helsinki and Helsinki University Hospital, Helsinki, Finland

**Keywords:** Methods, Cooling, Hypothermia, TTM, Cardiac arrest, Outcome

## Abstract

**Background:**

The aim of this study was to explore the performance and outcomes for intravascular (IC) versus surface cooling devices (SFC) for targeted temperature management (TTM) after out-of-hospital cardiac arrest.

**Methods:**

A retrospective analysis of data from the Time-differentiated Therapeutic Hypothermia (TTH48) trial (NCT01689077), which compared whether TTM at 33 °C for 48 h results in better neurologic outcomes compared with standard 24-h duration. Devices were assessed for the speed of cooling and rewarming rates. Precision was assessed by measuring temperature variability (TV), i.e., the standard deviation (SD) of all temperature measurements in the cooling phase. Main outcomes were overall mortality and poor neurological outcome, including death, severe disability, or vegetative status.

**Results:**

A total of 352 patients had available data and were included in the analysis; of those, 218 (62%) were managed with IC. A total of 114/218 (53%) patients with IC and 61/134 (43%) with SFC were cooled for 48 h (*p* = 0.22). Time to target temperature (≤ 34 °C) was significantly shorter for patients treated with endovascular devices (2.2 [1.1–4.0] vs. 4.2 [2.7–6.0] h, *p* < 0.001), but temperature was also lower on admission (35.0 [34.2–35.6] vs. 35.3 [34.5–35.8]°C; *p* = 0.02) and cooling rate was similar (0.4 [0.2–0.8] vs. 0.4 [0.2–0.6]°C/h; *p* = 0.14) when compared to SFC. Temperature variability was significantly lower in the endovascular device group when compared with SFC methods (0.6 [0.4–0.9] vs. 0.7 [0.5–1.0]°C; *p* = 0.007), as was rewarming rate (0.31 [0.22–0.44] vs. 0.37 [0.29–0.49]°C/hour; *p* = 0.02). There was no statistically significant difference in mortality (endovascular 65/218, 29% vs. others 43/134, 32%; *p* = 0.72) or poor neurological outcome (endovascular 69/218, 32% vs. others 51/134, 38%; *p* = 0.24) between type of devices.

**Conclusions:**

Endovascular cooling devices were more precise than SFC methods in patients cooled at 33 °C after out-of-hospital cardiac arrest. Main outcomes were similar with regard to the cooling methods.

## Introduction

Target temperature management (TTM) is recommended as an effective neuroprotective strategy in out-of-hospital cardiac arrest (OHCA) patients that remain comatose following the return of spontaneous circulations (ROSC), although this is based on low or very low level of evidence [1]. However, the benefits of cooling procedures in these patients remain controversial [2] and reduction of body temperature is neither easy nor without risk. Indeed, hypothermia can result in a decreased cardiac output and blood pressure, arrhythmias, increased risk of bleeding, hypokalemia, and increased insulin resistance [3]. Despite optimal target and duration of TTM have been investigated in large randomized clinical trials [4, 5], little is known about the optimal method to provide TTM to cardiac arrest patients.

TTM consists of different phases, i.e., induction, maintenance, rewarming, and fever control [3]. Over the last two decades, several cooling systems have been developed in order to achieve faster induction and more reliable temperature maintenance. In this setting, an ideal device should achieve target temperature quickly, allow for accurate maintenance and slow, controlled rewarming as well as avoid post-cooling fever. Initial methods to initiate TTM included body exposure, cooling pads or packs as well as the administration of intravenous cold fluids [6]; although being easy-to-use and cheap, these methods would produce unpredictable changes and variations in body temperature and increase the risk of frostbite and pulmonary edema [7]. More modern cooling devices, such as intravascular catheters (IC) or surface devices (SFC) with cold-water circulating blankets or hydrogel pads [8–10], provide a more rapid achievement of target temperature and a longer time within therapeutic temperature ranges (i.e., less overcooling and rebound hyperthermia), by the use of a temperature feedback control [11, 12].

Nevertheless, there may be significant differences in performance and adverse effects between SFC and IC [13]. Several studies have compared these two different cooling methods for TTM in post-anoxic brain injury [13–15]; however, most of them have limited cohorts of patients and no particular differences on patients’ outcome were observed between SFC and IC cooling techniques.

Thus, the aim of this study was to compare SFC methods with IC methods with regard to cooling precision, survival, neurological outcome, and adverse event among OHCA survivors.

## Methods

This is a post hoc analysis of data from the Time-differentiated Therapeutic Hypothermia (TTH48) trial (NCT01689077), a multicenter, randomized clinical trial conducted in Europe, which compared whether prolonged TTM at 33 °C for 48 h results in better neurologic outcome compared with standard 24-h duration [5]. The study protocol was approved by the Ethics Committees in each participating center, with written informed consent obtained from the next of kin or a legal surrogate before randomization. The study recruited 355 patients between February 2013 and June 2016 and demonstrated no significant difference in favorable neurologic outcome at 6 months for those treated during 48 h (69%) or 24 h (64%) of TTM.

Adult patients, resuscitated from OHCA of a presumed cardiac cause, older than 17 years and younger than 80 years, with sustained return of spontaneous circulation for more than 20 consecutive minutes, and with Glasgow Coma Scale (GCS) score less than 8, were included in the TTH48 trial. Exclusion criteria have been reported in the main manuscript [5]; for this study, we excluded those patients without data on the device used for TTM or without recording of hourly body temperature over the study period. All patients were sedated and treated with invasive mechanical ventilation. Other aspects of patient management were decided by the attending physician according to standard local practices.

During TTM, three periods were identified: (1) achievement of target temperature (time from initiation of cooling to first temperature < 34.0 °C), (2) maintenance of target temperature (time from target temperature to first temperature ≥ 34.0 °C), and (3) rewarming to 37.0 °C. Core temperature was mainly measured using urinary, esophageal, or intravascular probes. Temperature was managed with either SFC or IC methods, according to center preference, in combination with cold fluids to initiate TTM and rapidly reach the target temperature. After randomization, duration of cooling (i.e., 24 or 48 h) was considered from the time core temperature was 34 °C or lower. At the end of the 24- or 48-h period, rewarming was performed at a maximal rate of 0.5 °C/h until a core temperature of 37.0 °C was reached. Sedation was discontinued at 37.0 °C; the decision to keep devices on patients to avoid or minimize the occurrence of post-TTM fever accordingly was performed according to local practices.

Devices were assessed for (1) time from arrest to target temperature (i.e., < 34.0 °C), (2) time to target temperature (i.e., time from initiation of cooling to first body temperature < 34.0 °C), (3) cooling rate (i.e., changes in temperature from initiation of cooling to first body temperature < 34.0 °C, expressed as °C/h), (4) number of patients achieving the target temperature; (5) overcooling (i.e., at least one body temperature < 32.0 °C), (6) time spent outside targets (i.e., target is within 32 and 34 °C since the first body temperature < 34.0 °C until the initiation of rewarming; time outside target is expressed as number of hours or the percentage of hours according to the duration of cooling), (7) overshoot (i.e., body temperature after rewarming > 36.0 °C during cooling), (8) rewarming rate (i.e., changes in temperature between the initiation of rewarming to the first temperature > 37.0 °C, expressed as °C/h), and (9) post-TTM fever (i.e., number of patients with at least one body temperature measurement after rewarming exceeding 38.0 °C). Precision was assessed by measuring temperature variability (TV), i.e., the standard deviation (SD) of all temperature measurements in the cooling phase [16]. Main adverse events were collected throughout the hospital stay and reported as defined in the main trial [5]. Hyperglycemia was defined as a blood glucose > 150 mg/dL; hypernatremia was defined as serum sodium > 145 mEq/L; hypokalemia was defined as a serum potassium < 3.5 mEq/L.

Main outcomes were assessed at 180 days and included overall mortality and poor neurological outcome and defined a Cerebral Performance Categories score (CPC) of 3–5 (i.e., CPC 1 = alert, able to work and lead a normal life; CPC 2 = moderate cerebral disability and sufficient cerebral function for part-time work; CPC 3 = severe cerebral disability, dependent on others, and impaired brain function; CPC 4 = coma and vegetative state; CPC 5 = dead or certified brain dead).

### Statistical analysis

Statistical analyses were performed using IBM SPSS Statistics 24.0 for Windows. Descriptive statistics were computed for all study variables. A Kolmogorov–Smirnov test was used, and histograms and normal-quantile plots were examined to verify the normality of distribution of continuous variables. Data are presented as count (percentage) or median [25th–75th percentiles]. Differences between groups (i.e., SFC vs. IC) were assessed using a Fisher’s exact test for categorical variables and a Wilcoxon rank test for continuous variables. Data from repeated measures were analyzed using a two-way Friedman ANOVA and differences at each time point explored by the Dunn’s test. Multivariate regression analysis was performed to adjust overall mortality and unfavorable neurological outcome by the same predefined covariates (i.e., trial site, age, gender, initial cardiac arrest rhythm, time to return of spontaneous circulation, bystander-initiated life support, duration of cooling), as suggested in the main trial [5], as well as for those variables showing a statistical difference in the univariate analysis (*p* < 0.1—see Table 1). A *p* < 0.05 was considered as statistically significant.

**Table 1:** Characteristics of included patients, according to the cooling method. Data are expressed as count (%) or median (25th–75th percentiles)

	IC (*n* = 218)	SFC (*n* = 134)
Demographic characteristics		
Age, years	61 [53–53]	63.5 [55–70]^€^
Male gender, *n* (%)	189 (87%)	104 (78%)*
Weight, kg	85 [75–95]	80 [75–90]*
Previous neurologic disability, *n* (%)	6 (3%)	4 (3%)
Medical history		
Previous myocardial infarction, *n* (%)	30 (14%)	24 (18%)
Previous PCI or CABG, *n* (%)	27 (12%)	28 (21%)^€^
Previous cardiac arrest, *n* (%)	1 (0%)	2 (1%)
Chronic heart failure, *n* (%)	9 (4%)	9 (7%)
Chronic obstructive pulmonary disease, *n* (%)	13 (6%)	11 (8%)
Liver cirrhosis, *n* (%)	2 (1%)	1 (1%)
Chronic renal failure with dialysis, *n* (%)	2 (1%)	0 (0%)
Diabetes mellitus, *n* (%)	39 (18%)	24 (18%)
Immunocompromised, *n* (%)	3 (1%)	0 (0%)
Previous stroke, *n* (%)	13 (6%)	13 (10%)
Arrest characteristics		
Home location, *n* (%)	107 (49%)	85 (63%)*
Witnessed, *n* (%)	199 (91%)	124 (93%)
Bystander initiated CPR, *n* (%)	178 (82%)	115 (86%)
Shockable rhythm, *n* (%)	196 (90%)	116 (87%)
Time to return of spontaneous circulation, min	21 [15–30]	20 [16–26.8]
Mechanical chest compression, *n* (%)	56 (26%)	34 (25%)
Adrenaline, *n* (%)	135 (62%)	86 (64%)
Amiodarone, *n* (%)	93 (43%)	52 (39%)
Pre-ICU orotracheal intubation, *n* (%)	206 (94%)	137 (97%)
Pre-ICU cooling, *n* (%)	88 (40%)	59 (44%)
Coronary angiography, *n* (%)	184 (84%)	107 (80%)
PCI, *n* (%)	84 (39%)	61 (46%)
Characteristics on ICU admission		
Sedation, *n* (%)	216 (99%)	133 (99%)
Mean arterial pressure, mmHg	82 [69–96]	73.5 [63.3–84]
Lactate, mEq/L	2.8 [1.6–4.9]	2.5 [1.3–4.7]

*IC* intravascular catheter, *SFC* surface cooling, *PCI* percutaneous coronary intervention, *CABG* coronary artery bypass graft, *ICU* intensive care unit, *CPR* cardiopulmonary resuscitation. ^$^*p* < 0.001; *p < 0.05; ^€^*p* < 0.1

## Results

Of the 355 randomized to the trial, 3 were excluded because of lack of data on body temperature, leaving 352 (99%) patients for the final analysis. Of those, 218 (62%) were treated with IC and 134 (38%) with SFC. Also, 114/218 (53%) and 61/134 (46%) patients were cooled for 48 h using IC or SFC, respectively (*p* = 0.22). Main characteristics of the study population are reported in Table 1. Additional cooling methods were used in 75/218 (34%) patients in the IC group (*n* = 23 SFC; *n* = 67 cold fluids with 15 patients receiving both) and in 58/134 (42%, cold fluids—*p* = 0.11) in the SFC group.

**Fig. 1:**
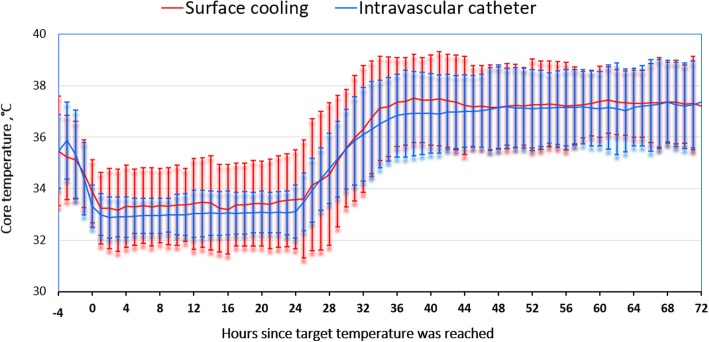
Temperatures in the intravascular catheter (IC) and surface cooling (SFC) groups until 72 h after achieving target temperature, with T0 defined as the time target temperature was reached. Duration of cooling = 24 h. Values are presented as mean ± 2 SDs

**Fig. 2:**
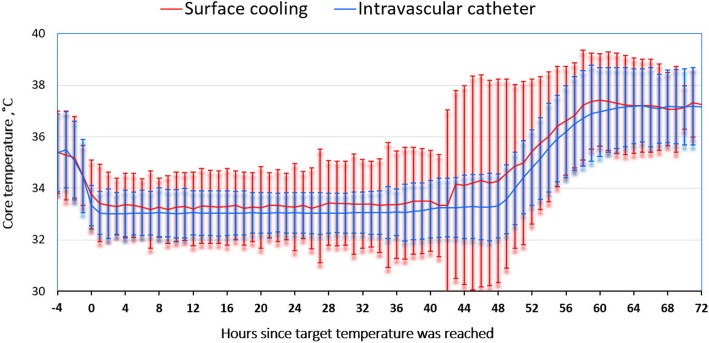
Temperatures in the intravascular catheter (IC) and surface cooling (SFC) groups until 72 h after achieving target temperature, with T0 defined as the time target temperature was reached. Duration of cooling = 48 h. Values are presented as mean ± 2 SDs

The mean ± SD temperatures for IC and SFC devices during the intervention are presented in Figs. 1 and 2, according to the duration of hypothermia. Main performance results are presented in Table 2. Time to target temperature was significantly shorter for patients treated with IC (2.2 [1.1–4.0] vs. 4.2 [2.7–6.0] h; *p* < 0.001), but temperature was also lower on admission (35.0 [34.2–35.6] vs. 35.3 [34.5–35.8] °C; *p* = 0.02) and cooling rate was similar (0.42 [0.18–0.81] vs. 0.34 [0.16–0.31]°C/h; *p* = 0.08) when compared with others. Temperature variability was significantly lower in the endovascular device group when compared with SFC methods (0.6 [0.4–0.9] vs. 0.7 [0.5–1.0]°C/h; *p* = 0.007), as was rewarming rate (0.31 [0.22–0.44] vs. 0.37 [0.29–0.49]°C/h; *p* = 0.002). The number of hours outside the therapeutic ranges was higher for the IC group, although the proportion of hours outside ranges on the total duration of cooling was similar between groups. Post-TTM fever was more frequent in the IC than in the SFC group.

**Table 2:** Performance findings on body temperature between the two groups. Data are expressed as count (%) or median (25th–75th percentiles). Number of hourly available temperature at normothermia = number of hourly recorded body temperature available after normothermia has been reached

	IC (*n* = 218)	SFC (*n* = 134)
Admission temperature, °C	35.0 [34.2–35.6]	35.3 [34.4–35.8]*
Time from Arrest to Temperature < 34 °C, hours	4.9 [3.9–6.5]	6.8 [5.1–9.0]^$^
Time to Temperature < 34 °C, hours	2.2 [1.1–4.0]	4.4 [2.8–7.0]^$^
First recorded Temperature < 34 °C, °C	33.8 [33.3–33.9]	33.6 [33.2–33.8]
Cooling Rate, °C/h	0.42 [0.19–0.81]	0.34 [0.16–0.61]
Duration of cooling, hours	40 [27–51]	28 [24–48] ^$^
During cooling		
- Mean temperature, °C	33.4 [33.1–33.8]	33.5 [33.2–33.8]
- Minimum temperature, °C	32.8 [32.5–33.0]	33.6 [32.3–32.9]*
- Maximal temperature, °C	35.2 [34.3–35.9]	35.5 [34.9–36.0]*
Temperature Variability during cooling, °C	0.65 [0.40–0.88]	0.69 [0.54–0.93]*
Patients never achieving target temperature, *n* (%)	16 (7)	6 (4)
Temperature outside targets, hours	7 [2–20]	5 [3–8]*
Temperature within targets, %	18 [5–36]	15 [7–27]
Patients with overcooling, *n* (%)	30 (14)	14 (10)
Patients with overshoot, *n* (%)	52 (24)	39 (29)
Early interruption of cooling, *n* (%)	8 (6)	6 (3)
Time to Normothermia, hours	9.3 [6.8–13.6]	7.5 [6.0–10.0]*
Rewarming Rate, °C/h	0.31 [0.22–0.44]	0.37 [0.29–0.49]*
Number of hourly available temperature at normothermia, hours	48 [24–81]	39 [18–54]
Max temperature reached during normothermia, °C	38.2 [37.8–38.6]	38.0 [37.6–38.4]
Post-TTM Fever, *n* (%)	137 (63)	67 (50)*

*IC* intravascular catheter, *SFC* surface cooling, *TTM* targeted temperature management. ^$^*p* < 0.001; *p < 0.05; ^€^*p* < 0.1

**Fig. 3:**
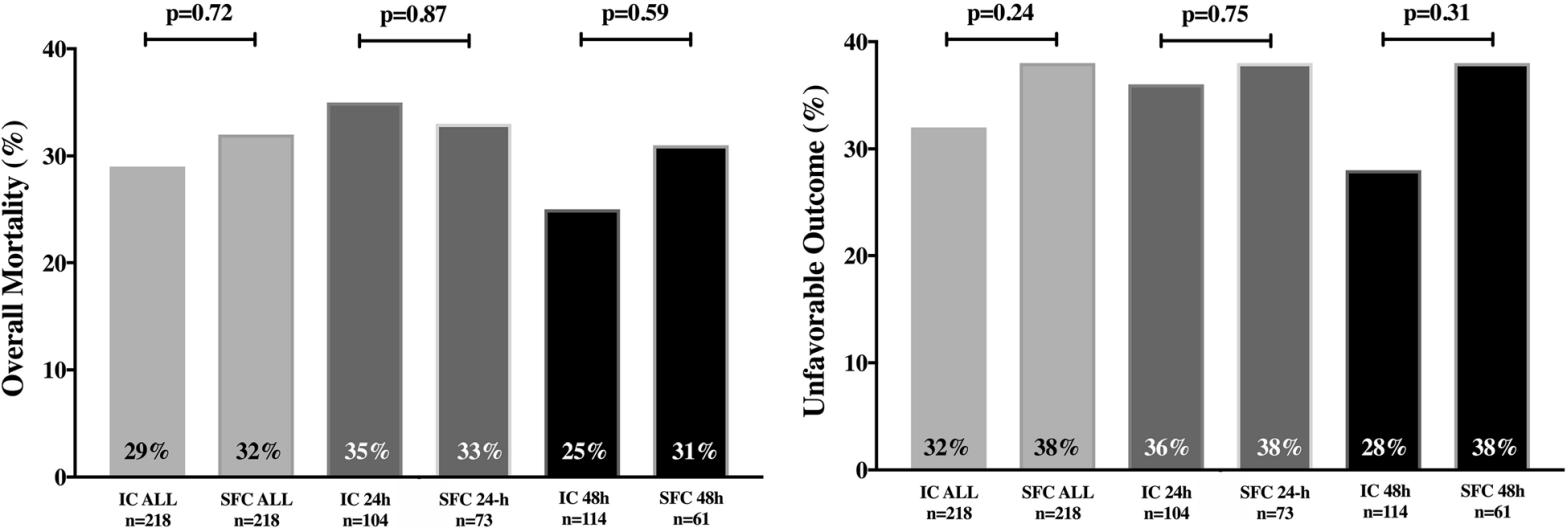
Mortality and unfavorable neurological outcome in the intravascular catheter (IC) and surface cooling (SFC) groups, according to the duration of cooling

There was no statistically significant difference in mortality (IC = 65/2178, 29% vs. SFC = 43/134, 32%; OR 0.89 [95% CIs 0.57–1.43], *p* = 0.65; adjusted OR 0.94 [0.48–1.81], *p* = 0.84) or poor neurological outcome (IC = 69/218, 32% vs. SFC = 51/134, 38%; OR 0.65 [0.33–1.25], *p* = 0.19; adjusted OR 0.78 [0.41–1.48], *p* = 0.45) between the type of devices, regardless of the duration of hypothermia (Fig. 3 and Table 3). The number of patients with at least one adverse event was similar between groups (Table 3); arrhythmias and hypokalemia were more frequent in the IC group, while hypernatremia was more frequent in the SFC group.

**Table 3:** Temperature analyses and outcomes of included patients, according to the methods of cooling. Data are expressed as count (%) or median (25th–75th percentiles)

	IC (*n* = 218)	SFC (*n* = 134)
Primary outcome		
CPC 3–5 at 6 months	69 (32%)	51 (38%)
Secondary outcomes		
Mortality at 6 months	65 (29%)	43 (32%)
Adverse events		
Any adverse event	203 (93%)	118 (88%)
Pneumonia	106 (49%)	56 (42%)
Other infections	75 (34%)	54 (40%)
Any bleeding	24 (11%)	16 (12%)
RBC transfusion	20 (9%)	15 (11%)
Seizure/myoclonus localized	27 (12%)	18 (13%)
Seizure/myoclonus globalized	39 (18%)	25 (19%)
Severe circulation failure	13 (6%)	15 (11%)^€^
Arrhythmias	105 (48%)	50 (37%)*
Severe (VT/VF or unstable despite treatment)	21 (10%)	19 (14%)
New cardiac arrest requiring CPR	7 (3%)	5 (4%)
Renal replacement therapy	13 (6%)	14 (10%)
Hyperglycemia	169 (78%)	113 (83%)
Hypernatremia	21 (10%)	24 (18%)*
Hypokalemia	88 (40%)	38 (28%)*
Resource use		
ICU length of stay (days)	5 [3–7]	6 [3–9]

*IC* intravascular catheter, *SFC* surface cooling, *RBC* red blood cells. ^$^*p* < 0.001; *p < 0.05; ^€^*p* < 0.1

## Discussion

In this study evaluating the database of a randomized clinical trial, we evaluated the differences in temperature control between IC and SFC methods to achieve TTM among survivors of OHCA. The use of IC was associated with a similar time to target temperature (i.e., speed of cooling), a lower temperature variability during the maintenance phase, a slower rewarming rate, but more frequent post-TTM fever than SFC devices. No differences in mortality or poor neurological outcome between the two methods were observed. Also, the rate of adverse events was similar between groups.

The optimal cooling technique to deliver TTM after OHCA remains still unknown. Although international guidelines recommend to use devices with a continuous temperature feedback control (TFC) in this setting, to minimize the risk of overcooling and to provide a more stable control of target temperature [17], invasive methods using IC with TFC are the most precise cooling techniques to provide TTM, even when compared to external SFC devices [13]. However, whether this might influence patients’ outcome, it is still debated. The choice of one or the other method is usually driven by different considerations such as precision, workload for nurses, costs, and the risk/benefit ratio. External SFC methods are applied around the torso and limbs and can adequately induce and maintain TTM in OHCA patients [18]; however, they also require more nursing attention, are frequently associated with shivering, may reduce overall access to the patient, and induce some skin damage [19]. Intravenous cooling with IC devices are effective for TTM in OHCA patients, but they need a trained physician to insert the catheter into the large vein and may be associated with a higher risk of infection and bleeding [19].

Assessment of IC and SFC devices has already been performed in large animal models, showing a faster cooling rate for IC when compared with others [20]. In humans, results are more controversial. Ferreira et al. showed that the time to target temperature was faster and the rewarming rate lower in IC-treated patients when compared with the SFC group [21]. On the opposite, Tømte et al. observed no differences in cooling rates, post-TTM fever, and the occurrence of main adverse events between SFC and IC cooling methods [22]. Similar results on cooling rate and the occurrence of overcooling were observed in another study, although target temperature maintenance was more stable in the IC group than in SFC-cooled patients [23]. Less fluctuation of body temperature during the maintenance phase with the use of IC devices when compared with SFC methods was observed also in other studies [7, 24]. In our study, the cooling rates were similar between groups. However, there was a higher number of patients in the SFC group receiving also cold fluids; administration of cold fluids is actually the most common and rapid method to induce hypothermia, especially when administered in the pre-hospital setting, although some concerns on its effectiveness and potential harms exist [25], and may have contributed to faster-cooling rates in the SFC group. We observed a lower TV in the IC-treated patients than in the SFC group. Despite this finding might again underline a higher precision to maintain the target temperature using IC, TV was somewhat higher in patients with favorable than unfavorable neurologic outcome [16] and might suggest intact thermoregulatory pathways that aim to restore a body temperature close to 37.0 °C rather than a target to optimize TTM. The difference in TV was so limited (< 0.1 °C) between groups that one may argue whether this can translate in clinically relevant benefits on patients’ outcome. Moreover, the proportion of time outside therapeutic ranges was similar between groups, suggesting that despite a more stable core temperature, the effectiveness of the devices to maintain the body temperature in the selected target was similar. Finally, rewarming rate was lower in the IC group, although the difference was numerically small (i.e., 0.06 °C). There are no clinical data suggesting a critical cut-off for harm of excessively rapid rewarming after TTM in OHCA survivors, although high rewarming rate, exceeding 0.5 °C, might be associated with a higher risk of poor neurological outcome (71% vs. 52%) than slower rewarming [26]. As both cooling devices in our study had rewarming rate below this cut-off of 0.5C°/h, it is hard to conclude that the minimal differences in rewarming rate observed in this cohort can result in any clinically relevant result.

Previous studies have also reported more frequent complications with the IC than with SFC devices, although these findings remain discordant and might be due to chance or selection bias among different studies. In one study, infectious and cardiovascular complications were similar between IC and SFC [7]. In a large cohort, shivering, electrolyte disturbances, and arrhythmias were also similar between IC and SFC devices [15]. In a small RCT, pneumonia and ischemic stroke were more frequent in the SFC group, while the occurrence of significant arrhythmias and renal failure was higher in the IC group [11]. In a matched-control analysis, the use of IC was associated with a greater incidence of sepsis [27]. In another study, an increase in bleeding was observed in the IC group (14% versus 2%, *p* = 0.11) [14]. We also observed relatively similar occurrence of adverse events between IC and SFC devices in our study, except for more frequent arrhythmias and hypokalemia in the IC group, probably due to a larger proportion of patients being cooled for 48 h. Also, post-TTM fever was more frequent in the IC group, which is in contrast with previous publications [22, 23]. These findings might be potentially explained either by a higher number of recorded temperatures in the IC group (i.e., more data available which translates in a higher probability to detect fever) or by the early removal of the endovascular system, because of the risk of infection and bleeding, which would have exposed these patients to a less accurate temperature control in the post-cooling phase. Importantly, as no specific data on shivering control, sedation policies, and/or catheter removal were reported, we could not specifically analyze the determinants of post-TTM fever in this cohort. However, the safety profile of both cooling systems looks comparable, and the selection of one strategy over the other might be influenced by the potential benefits on mortality and neurological recovery.

Together with some advantages in the TTM delivery for IC over SFC devices and a similar safety profile, no significant differences in patients’ outcome were observed between groups. Whether our study is simply underpowered to show any statistical significance between the methods or if this difference is related to an imbalance between IC and SFC (i.e., more patients cooled for 48 h, less arrest occurring at home, and a less significant difference in outcome when adjusted for confounders), it is impossible to conclude from our data. In a recent post hoc analysis of a large RCT including 934 patients [15], mortality and poor neurological outcome was lower in the IC group when compared with SFC devices (46.3% vs. 50.0% and 49.0% vs. 54.3%, respectively). Other studies also showed a non-significant reduction in mortality or poor neurological outcome in the IC group of around 5–10% [7, 14, 22, 23], although this was not consistent in another study [27, 28]. Importantly, this might be explained by chance, selection bias or by different case-mixes (i.e., large referral centers with PCI facilities tend to use endovascular cooling techniques). Moreover, SFC devices are not entirely comparable as they include a wide range of devices, from simple ice bags to sophisticated machines with automatic TFC using blankets containing circulating coolant, and these differences may also impact on outcome in this setting. In one study, Shinada et al. observed that the use of SFC with self-adhesive, hydrogel-coated pads gel-circulating and TFC was associated with a lower mortality and poor neurological outcome (20% vs. 27% and 28% vs. 45%, respectively) than conventional SFC using blankets [18]. In another small RCT, Heard et al. showed that hydrogel-coated pads gel-circulating and TFC had a lower proportion of patients with poor neurological outcome (39% vs. 47%) than the use of cooling blankets and ice [11]. As such, future studies comparing IC and SFC with TFC are needed to better understand the impact of such cooling strategies on patients’ outcome. Also, a systematic review and meta-analysis of the existing literature might help to further quantify the relevance of cooling methods on mortality and neurological outcome of OHCA survivors.

This study has also several limitations. First, selection bias related to the use of different cooling methods in the different participating centers may account for some of the differences between groups. In particular, it is impossible to consider whether a real choice or equipoise existed between IC and SFC at the time point of treatment selection for those centers where both devices were available. Moreover, some centers had only of the two devices available so that differences in outcome, despite similar cooling times, might be influenced by the “site” effect, although this was considered into the adjusted analysis. Second, the study was not powered to detect differences in clinical outcome. Third, the accuracy of intervals, such as time to cooling or target temperature, might be unreliable since this information may be subject to reporting or measurement errors. Forth, different sites of temperature measurement were used, which may have led to a measurement bias. Also, brain temperature typically exceeds body temperature by 0.5 to 2.0 °C after an acute brain injury, so that measuring peripheral temperature is a poor indicator of temperature control within the cerebral tissue [28]. Fifth, this was not a randomized study primarily investigating performance of cooling devices; as such, the risk of bias is high and any association found should be cautiously interpreted. Also, we did not report about the type of devices (i.e., different types of endovascular devices or the proportion of cold-water circulating blankets vs. hydrogel pads in the surface cooling group), and this may contribute to a significant variability in TTM effectiveness and precision within the groups. Moreover, some differences between groups (i.e., arrest location) were not considered in the adjusted analysis of outcomes. Finally, we have analyzed cooling devices by categories rather than comparing individual IC or SFC devices and the heterogeneity among different systems can also account for some observed differences between groups.

## Conclusions

In this study, endovascular cooling devices were more precise than SFC methods in patients cooled at 33 °C after out-of-hospital cardiac arrest. Main outcomes (i.e., mortality and neurological outcome) were similar with regard to the cooling methods, which suggest no clinically relevant differences in this setting.

## Abbreviations

CPC: Cerebral Performance Category
GCS: Glasgow Coma Score
IC: Intravascular catheter
OHCA: Out-of-hospital cardiac arrest
RCT: Randomized clinical trial
ROSC: Return of spontaneous circulation
SFC: Surface cooling
TFC: Temperature feedback control
TTM: Targeted temperature management
TV: Temperature variability
